# Proteasomal inhibition sensitizes cervical cancer cells to mitomycin C-induced bystander effect: the role of tumor microenvironment

**DOI:** 10.1038/cddis.2015.292

**Published:** 2015-10-22

**Authors:** S V Singh, A K Ajay, N Mohammad, P Malvi, B Chaube, A S Meena, M K Bhat

**Affiliations:** 1 National Centre for Cell Science, Savitribai Phule Pune University Campus, Ganeshkhind, Pune 411007, India

## Abstract

Inaccessibility of drugs to poorly vascularized strata of tumor is one of the limiting factors in cancer therapy. With the advent of bystander effect (BE), it is possible to perpetuate the cellular damage from drug-exposed cells to the unexposed ones. However, the role of infiltrating tumor-associated macrophages (TAMs), an integral part of the tumor microenvironment, in further intensifying BE remains obscure. In the present study, we evaluated the effect of mitomycin C (MMC), a chemotherapeutic drug, to induce BE in cervical carcinoma. By using cervical cancer cells and differentiated macrophages, we demonstrate that MMC induces the expression of FasL via upregulation of PPAR*γ* in both cell types (effector cells) *in vitro*, but it failed to induce bystander killing in cervical cancer cells. This effect was primarily owing to the proteasomal degradation of death receptors in the cervical cancer cells. Pre-treatment of cervical cancer cells with MG132, a proteasomal inhibitor, facilitates MMC-mediated bystander killing in co-culture and condition medium transfer experiments. In NOD/SCID mice bearing xenografted HeLa tumors administered with the combination of MMC and MG132, tumor progression was significantly reduced in comparison with those treated with either agent alone. FasL expression was increased in TAMs, and the enhanced level of Fas was observed in these tumor sections, thereby causing increased apoptosis. These findings suggest that restoration of death receptor-mediated apoptotic pathway in tumor cells with concomitant activation of TAMs could effectively restrict tumor growth.

Owing to the heterogeneous nature and scanty vascularization, the access of anticancer regimen to all strata of the tumor is one of the major challenges in cancer therapy. Current response rate to chemotherapy is far from desirable and warrants formulating the strategies to enhance specificity and efficacy of the anticancer regimens. Of late, the phenomenon of bystander effect (BE), which refers to transmission of death signals from the drug-exposed cells to the unexposed cells, is being explored to improve the therapeutic response. Although BE has been well documented in radiotherapy^1, 2^ and experimental approaches of gene therapy,^3, 4^ very limited information is available with respect to conventional chemotherapeutic drugs. We have previously demonstrated the chemotherapy-induced bystander killing in breast cancer cells^5^ and hepatocellular carcinoma cells.^6^ Recently, other groups also have demonstrated the occurrence of chemotherapy-induced BE in breast cancer^7^ and lung cancer,^8, 9^ which is in agreement with our studies. BE has been shown to be dependent on cell type and class of drugs,^6^ and the role of tumor microenvironment in response to chemotherapeutic drug-induced BE is poorly understood.

Cervical cancer is one of the most common solid tumors. Mitomycin C (MMC), a DNA alkylating agent, has been widely used in this malignancy as a constituent of combination therapy.^10^ From the pharmacological point of view, MMC is effective at relatively low dose with minimal organ-associated toxicity^11^ and it has been shown to activate innate immunity.^12^ However, therapeutic efficacy of MMC principally depends on other drug types in combination therapy.^13^ Therefore, a well-designed strategy that could enhance the efficacy of MMC is desirable. MMC has been demonstrated to induce BE in hepatocellular carcinoma, but not in cervical cancer cells.^6^ Although the precise mechanisms of bystander killing remain elusive, we have previously reported the involvement of death ligands,^5, 6^ which was later supported by other studies.^7, 8, 9^ The ability of cancer cells to escape programmed cell death has a critical role in the survival of cancer cells and tumor progression. Despite the presence of cellular apoptotic factors, cancer cells reprogram their molecular events and signaling to evade apoptosis.^14^ Moreover, it has been reported that exposure to proteasomal inhibitor inhibits the growth of various cancer cells and sensitize them to death ligand-mediated death by stabilizing death receptors.^15, 16, 17^ Considering these notions, we speculated that non-functionality of death receptors could be one of the possible factors associated with defective BE in cervical cancer. We, therefore, hypothesized that treating cervical cancer cells with combination of MMC and proteasomal inhibitor could elicit BE, and thereby may significantly improve the therapeutic outcome.

Till date, studies explicate cancer cells exposed to chemotherapy as the effector cells in inducing bystander-mediated killing. However, owing to the heterogeneous nature of cellular population in tumor, other cellular components are also likely to have a key role in inducing BE. Tumor microenvironment consists of a heterogeneous mass of malignant as well as nonmalignant cells. The nonmalignant cells include endothelial, fibroblast and immune cells that establish multitude of interactions among themselves and also with malignant cells.^9^ Macrophages are the most abundant immune cells present in tumors, also termed as tumor-associated macrophages (TAMs).^18^ TAMs are differentiated monocytes that infiltrate the tumor microenvironment, and are exposed to chemotherapeutic regimen. Studies have demonstrated that TAMs could account for approximately more than 60% of tumor mass in some cancers.^19, 20, 21^ TAMs exposed to radiations^2^ and chemotherapy^22^ have been shown to have a significant role in inducing BE. Studies support the notion that targeting TAMs could improve the therapeutic index of various drugs.^10, 23^ Increased sensitivity to cyclophosphamide^14^ and cisplatin^24^ has been shown in co-culture system involving cancer cells and macrophages. Under chemotherapy, increased recruitment of macrophages with enhanced expression of tumoricidal factors like perforin and granzyme,^22^ death ligands^10^ or ROS ^25^ has been reported in tumors. Therefore, we speculated that BE could further be amplified by infiltrating macrophages resulting in enhanced therapeutic efficacy of anticancer regimens. In the present study, we evaluated combination effect of MMC and MG132 in enhancing bystander killing of cancer cells *in vitro* and *in vivo*, in part, through the involvement of cancer cells and TAMs. Herein, we demonstrate that stabilization of Fas on cervical cancer cells facilitates dramatic reduction in tumor progression as a consequence of increase in apoptosis. This study could be helpful in designing novel therapeutic strategies in treating cancer by involving proteasomal inhibitors in combination with chemotherapeutic drugs that specifically activate death receptor-mediated killing.

## Results

### MMC induces FasL expression in effector cells

**Figure 1:**
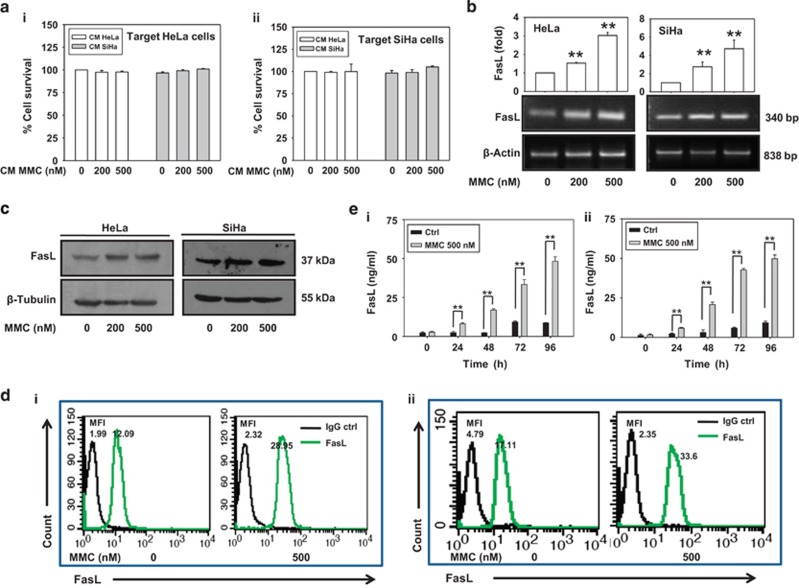
MMC induces expression of death ligands in cervical cancer cells. (**a**) Bystander killing in CM transfer experiment. The effector cells (HeLa and SiHa) were treated with indicated concentrations of MMC for 24 h, and then CM medium was collected after 48 h as described in Materials and Methods. Target HeLa (i) and SiHa (ii) cells were incubated with the respective CM for 24 h. Cell survival was evaluated by MTT assay. (**b**) Semi-quantitative RT-PCR for FasL mRNA. HeLa and SiHa cells were treated with indicated concentrations of MMC for 24 h, and were processed for RT-PCR. *β*-Actin was used as a loading control. Data are mean±S.D., and are representative of three independent experiments. (**c**) Western blot analysis of FasL. HeLa and SiHa cells were treated with indicated concentrations of MMC for 24 h, and cell lysates were subjected to SDS-PAGE and probed for protein levels of FasL. (**d**) Flow cytometric analysis of FasL expression. HeLa (i) and SiHa (ii) cells were treated with MMC as described above. Untreated or MMC-treated cells were probed with FasL primary antibody or IgG control (1 : 100), and further with PE-conjugated secondary antibody (1 : 200). Cells were then washed with PBS, and FasL expression was analyzed by flow cytometry. (**e**) Sandwich ELISA for quantification of sFasL from MMC-treated HeLa (i) and SiHa (ii) cells at the indicated time points. Data are mean±S.D., and are representative of three independent experiments (**P<0.01 when compared with their respective controls)

We have previously demonstrated that MMC induces bystander killing in hepatocellular carcinoma cells.^6^ However, MMC failed to promote bystander killing in cervical cancer cells under identical experimental conditions (Figures 1ai and aii). This led us to further investigate the ability of MMC to differentially promote bystander response in other cancer cell types. We examined whether MMC could induce expression of death ligands in cervical cancer (HeLa and SiHa) cells. After 24 h of treatment with MMC, FasL was increased in dose-dependent manner at transcriptional as well as protein levels (Figures 1b and c), but TRAIL expression remained unchanged (Supplementary Figure 1a). However, treatment with other anticancer drugs such as cisplatin, 5-fluorouracil, carboplatin and paclitaxel did not affect FasL expression in HeLa cells significantly (Supplementary Figure 1b). As FasL is a secretory as well as membrane-bound protein, we checked the expression of FasL on the cell surface following MMC treatment. Flow cytometric analysis confirmed that MMC-induced FasL expression on membrane of HeLa and SiHa cells (Figures 1di and dii). Consistent with these observations, secretory FasL (sFasL) level was also increased in the conditioned medium (CM) collected from cells treated with MMC in a time-dependent manner (Figures 1ei and eii).

**Figure 2:**
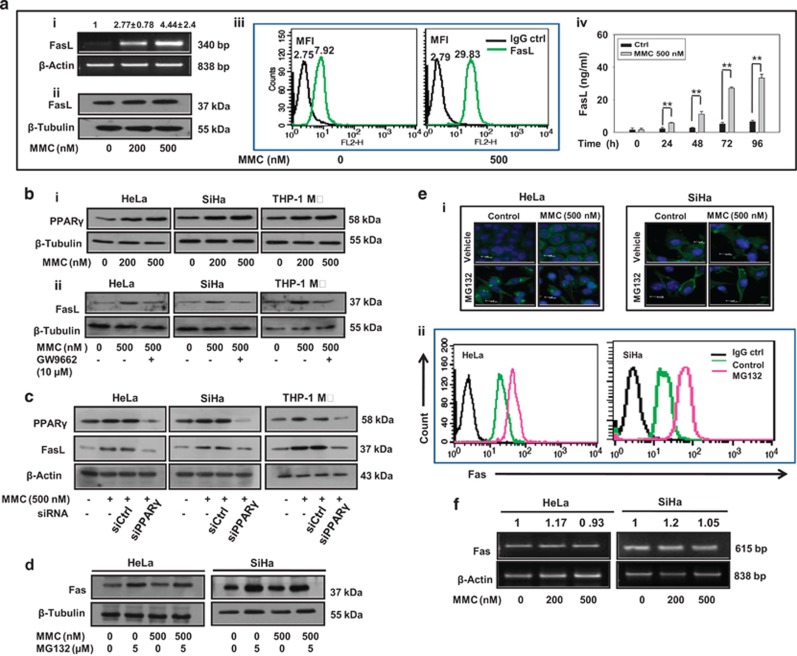
MMC induces death ligand expression via PPAR*γ* and proteasomal inhibition increases level of death receptors in cervical cancer cells. (**a**) Analysis of expression of FasL in MMC-treated THP-1 MΦ. (i) Semi-quantitative RT-PCR for FasL mRNA. THP-1 MΦ were treated with indicated concentrations of MMC, and processed for RT-PCR. *β*-Actin was used as a loading control. Data are mean±S.D., and are representative of three independent experiments. (ii) THP-1 MΦ were treated with indicated concentrations of MMC, and whole-cell lysate were subjected to western blotting for FasL. (iii) Flow cytometric analysis of FasL expression. THP-1 MΦ were treated with MMC as described above. Untreated or MMC-treated cells were probed with FasL primary antibody or IgG control (1 : 100), and further with PE-conjugated secondary antibody (1 : 200). Cells were then washed with PBS, and FasL expression was analyzed by flow cytometry. (iv) Sandwich ELISA for quantification of sFasL from untreated and MMC-treated THP-1 MΦ at indicated time points. (**b**) Analysis of involvement of PPAR*γ* in the regulation of FasL expression. (i) Western blot analysis of PPAR*γ* in MMC-treated cells. HeLa, SiHa and THP-1 MΦ were plated in 35 mm culture dishes. After 24 h, MMC treatment was given at indicated concentrations, and cells were further incubated for 24 h. Cell lysates were then subjected to SDS-PAGE and western blotting for PPAR*γ*. (ii) Effect of PPAR*γ* inhibition on FasL expression. HeLa, SiHa and THP-1 MΦ were plated in 35 mm culture dishes. After 24 h, cells were pretreated with GW9662 (10 *μ*M) for 2 h. Thereafter, MMC (500 nM) treatment was given and cells were incubated for 22 h. Whole-cell lysates were subjected to western blotting for FasL. (**c**) Effect of knockdown of PPAR*γ* on MMC-induced expression of FasL. HeLa, SiHa and THP-1 MΦ were transfected with control siRNA or PPAR*γ* siRNA for 15 h, and allowed to grow for a further 15 h. Control siRNA and PPAR*γ* siRNA-transfected cells were exposed to MMC for 24 h, and cells were collected for western blot analysis of PPAR*γ* and FasL. (**d**) MG132-induced expression of Fas. HeLa and SiHa cells treated with MMC and/or MG132 for 24 h. Western blot analysis of whole-cell lysates subjected to SDS-PAGE and probed for Fas. (**e**) Analysis of expression and localization of Fas in MG132-treated cervical cancer cells. (i) Immunofluorescence staining of HeLa and SiHa cells. Cells were treated with MMC and/or MG132 for 24 h, washed twice, then fixed and permeabilized with 4% paraformaldehyde and 1% Triton X-100 respectively, and blocked with 5% FBS. Cells were further incubated with anti-Fas primary antibodies (1 : 100) for 2 h and subsequently stained with FITC-conjugated secondary antibodies (1 : 200) for 1 h. (ii) Flow cytometric analysis of Fas expression in cervical cancer cells. HeLa and SiHa cells were treated with MG132 for 24 h. Untreated or MG132-treated cells were probed with primary antibody against Fas or IgG control (1 : 100) for 1 h, and further with PE-conjugated secondary antibody (1 : 200) for 30 min. Cells were then washed with PBS, and Fas expression was analyzed by flow cytometry. (**f**) Semi-quantitative RT-PCR for Fas mRNA in MMC-treated cervical cancer cells. HeLa and SiHa cells were treated with indicated concentrations of MMC for 24 h, and were processed for RT-PCR. *β*-Actin was used as a loading control

Tumor microenvironment has a crucial role in defining the efficacy of a chemotherapeutic drug.^10^ TAMs, the differentiated infiltrating monocytes exposed to the onslaught of the drugs, may also be involved in bystander killing. To understand the role of tumor microenvironment in BE, we explored the expression of FasL in THP-1 monocytes/macrophages in response to MMC. Consistent with findings in effector cervical cancer cells, we found that exposure of THP-1 monocytes/macrophages to MMC caused increase in FasL expression in a concentration-dependent manner (Supplementary Figure 1c; Figures 2ai and aii). The flow cytometric analysis also confirmed the MMC-induced expression of membrane-FasL on THP-1 macrophages (THP-1 MΦ Figure 2aiii). In addition, concentration of sFasL was markedly increased in CM of MMC-treated effector cells as compared with control in a time-dependent manner (Figure 2aiv). Surprisingly, despite increased cell surface as well as sFasL concentration in response to MMC, no cell death was observed in the target cervical cancer cells.

### PPAR*γ* mediates MMC-induced FasL expression

We probed into the possible upstream factors responsible for enhanced expression of FasL upon MMC treatment and narrowed down to the role of PPAR*γ*, a transcription factor reported to regulate FasL expression.^26, 27^ We found that MMC treatment enhanced PPAR*γ* level in a dose-dependent manner in cervical cancer cells as well as in macrophages (Figure 2bi). Further, to confirm the role of PPAR*γ*, effector cells (HeLa, SiHa and THP-1 MΦ) were treated with MMC in the presence of GW9662, an antagonist of PPAR*γ* (Figure 2bii) or cells transfected with PPAR*γ*-specific siRNA (Figure 2c). In both the cases, FasL expression was diminished as compared with MMC alone treated cells, collectively suggesting involvement of PPAR*γ* in MMC-induced FasL expression.

### Proteasomal inhibition enhances susceptibility of cervical cancer cells to MMC-induced FasL-mediated killing

**Figure 3:**
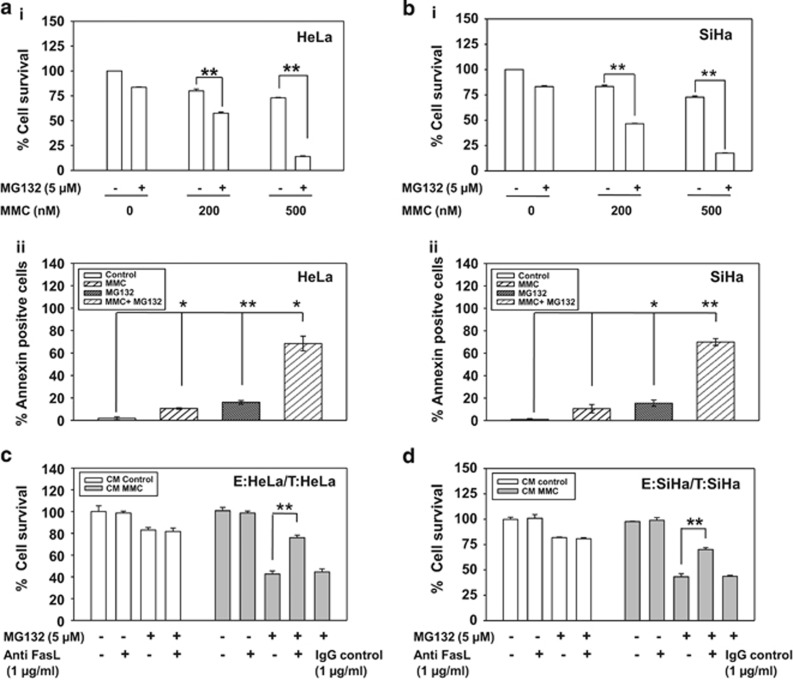
MG132 sensitizes cervical cancer cells to FasL-mediated cell death. (**a**i and **b**i) MTT assay in MG132 and MMC-treated HeLa and SiHa cells. HeLa (**a**i) and SiHa (**b**i) cells (7 × 10^3^/well) were seeded in 96-well plates. After 24 h, treatment of 5 *μ*M MG132 was given for 2 h. Thereafter, 200 or 500 nM MMC was added for next 34 h, and MTT assay was performed. (**a**ii and **b**ii) Annexin V-FITC staining in MG132 and MMC-treated cervical cancer cells. HeLa (**a**ii) and SiHa (**b**ii) cells (1 × 10^5^) were seeded in 35 mm plate. After 24 h, treatment of 5 *μ*M MG132 was given for 2 h. Thereafter, 500 nM MMC was added for further 34 h. Next, cell were stained with annexin V-FITC, and analyzed by flow cytometry. Data are mean±S.D., and are representative of three independent experiments (*P<0.05, **P<0.01 when compared with their respective controls). (**c** and **d**) Involvement of sFasL in mediating bystander cytotoxicity. The effector cells (HeLa and SiHa) were treated with 500 nM MMC for 24 h and then CM medium was collected after 48 h as described in Materials and Methods. Target HeLa (**c**) and SiHa (**d**) cells were incubated with the respective CM in the presence or absence of MG132 and/or anti-FasL antibody or IgG control antibody for 36 h. CM was supplemented with 0.2% FBS to avoid cell death owing to growth factor depletion. Cell survival was evaluated by MTT assay. Data are mean±S.D., and are representative of three independent experiments (*P<0.05, **P<0.01, when compared with their respective controls)

Despite induced expression of FasL, the reasons for non-occurrence of bystander killing following MMC treatment, was further investigated. HPV infection is reported to cause activation of proteasomal degradation pathway in cancer cells.^28^ It has also been reported that MG132 sensitizes multiple myeloma^29^ and other cancer cells^17, 30^ to death ligand-mediated apoptosis. Interestingly, we found that MG132 enhanced Fas expression in HeLa and SiHa cells (Figure 2d). Enhanced localization of Fas to the plasma membrane was observed by confocal microscopy (Figure 2ei) and FACS analysis (Figure 2eii), which was sustained up to 24 h even after the withdrawal of MG132, as detected by confocal microscopy (Supplementary Figure 1d). However, MMC treatment did not affect the expression of Fas at protein and mRNA level (Figures 2d and f). We therefore evaluated the combination effect of MMC and MG132 on HeLa and SiHa cells. In cell survival assay, it was found that MMC treatment, when combined with MG132, diminished the survival in a dose-dependent manner as compared with either treatment alone in HeLa and SiHa cells (Figures 3ai and bi). Under identical experimental setup, we also observed significant increase in annexin V-FITC positive cells in combination treatment as compared with either agent alone (Figures 3aii and bii).

Toillon *et al.*^31^ and Chippa *et al.*^5^ have reported that low expression level of Fas parallels with resistance to FasL-mediated killing in MDA-MB-231 cells. We detected higher expression of Fas on the cell membrane in MCF-7 cells compared with MDA-MB-231 cells (Supplementary Figure 2), which correlates with FasL-mediated cell death. Next, to investigate whether MG132 could sensitize cervical cancer cells to MMC-induced bystander killing, CM was collected from effector cells treated with MMC, and the target cells were incubated with this CM in the presence or absence of MG132. We observed that MG132 treatment enhances death in target cells grown in CM from MMC-treated effector cells compared with control CM (Figures 3c and d). Further, to confirm the involvement of FasL in bystander killing, we supplemented FasL-neutralizing antibody in the CM collected from MMC-treated effector cells, which significantly diminished killing in bystander target cells in the presence of MG132 (Figures 3c and d). In the presence of MG132, MMC-concentration-dependent killing of bystander target cells was detected (Supplementary Figures 3a and b), which parallels with the expression level of FasL.

**Figure 4:**
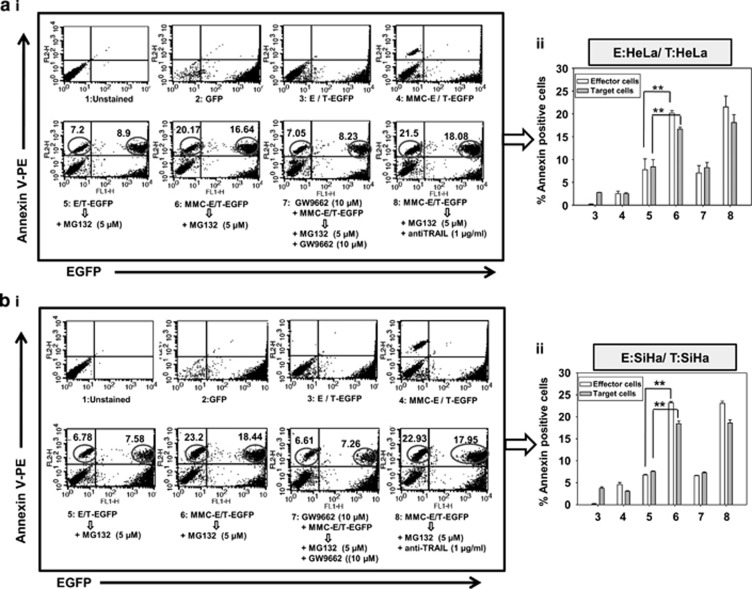
Co-plating experiments to evaluate contact-dependent bystander killing in homogeneous system. Analysis of apoptotic cell death in target EGFP-expressing cervical cancer cells. (**a**i) Histograms for effector (HeLa) and target cell (HeLa-EGFP) populations alone or in co-culture are shown after annexin V-PE staining as described in Materials and Methods. 1: Untreated HeLa cells; 2: Untreated HeLa-EGFP cells; 3: Untreated effector cells were co-plated with target EGFP cells; 4: Effector cells were treated with MMC for 24 h, washed with medium and target EGFP cells were co-plated; 5: Untreated effector cells were co-plated with target EGFP cells and treated with MG132 for 24 h; 6: Effector cells were treated with MMC for 24 h, washed with medium, and target EGFP cells were co-plated and then treated with MG132 for 24 h; 7: Effector cells were treated with GW9662 for 2 h followed by the addition of MMC, for 24 h, and then washed, co-plated with target EGFP cells along with treatment of MG132 and GW9662; 8: Effector cells were treated with MMC for 24 h, washed and co-plated with target EGFP cells, and further incubated in the presence of MG132 and anti-TRAIL antibodies; are shown with annexin V-PE positive counts and are indicated as percentages of apoptotic cells. Upper right quadrant represents proportion of apoptotic target EGFP cells. (**a**ii) The bar graph shows annexin V-PE positive cells in the same experiment. Data are mean±S.D., and are representative of three independent experiments (**P<0.01, when compared with their respective controls). (**b**i) Similar experiment was performed in SiHa cells (effector SiHa and target SiHa-EGFP). (**b**ii) The bar graph shows annexin V-PE positive cells in the same experiment. Data are mean±S.D., and are representative of three independent experiments (**P<0.01, when compared with their respective controls)

In addition to the involvement of secretory factors, BE is also exhibited in a contact-dependent manner. Therefore, to verify this phenomenon, we exploited the advantage of using EGFP-expressing target cells to measure the cell death specifically in target cells. For this, phycoerythrin (PE)-conjugated annexin V staining in a homogeneous co-culture of the effector cell (HeLa) and target cells (HeLa-EGFP) was quantified. In FACS analysis, higher percentage of annexin V-PE-positive target cells were detected in co-culture system involving MMC-treated effector cells co-plated with target EGFP cells, in the presence of MG132 compared with either conditions. Moreover, the bystander killing was significantly diminished in the presence of PPAR*γ* inhibitor GW9662 indicating involvement of PPAR*γ* in regulating MMC-induced bystander killing via FasL. However, no effect of TRAIL-neutralizing antibody on cell killing was observed implying its non-involvement in this phenomenon (Figures 4ai and 4aii). Similar experiments were performed with SiHa cells and findings were consistent with those in HeLa cells (Figures 4bi and bii).

**Figure 5:**
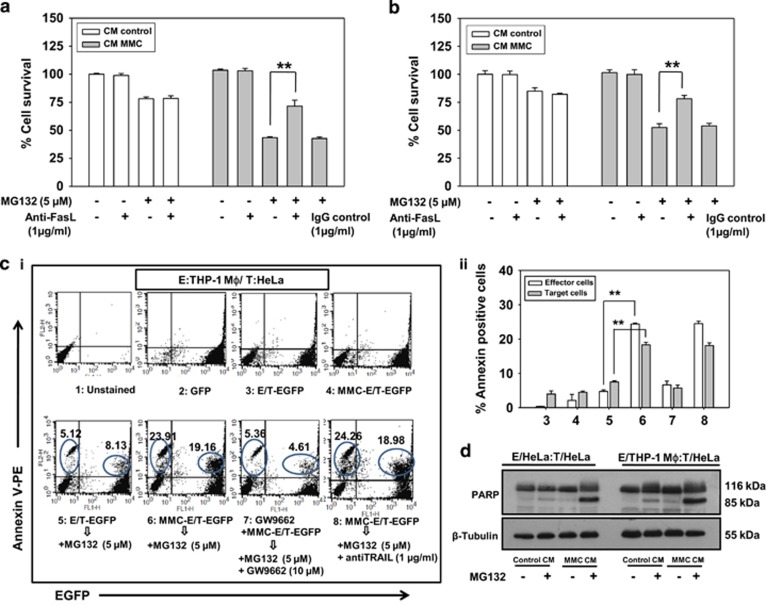
CM transfer and co-plating experiments to evaluate bystander killing in heterogeneous system. Involvement of sFasL in mediating bystander cytotoxicity. The effector cells (THP-1 MΦ) were treated with 500 nM MMC for 24 h, and then CM was collected after 48 h as described in Materials and Methods. Target HeLa (**a**) and SiHa (**b**) cells were incubated with the CM in the presence or absence of MG132 and/or with anti-FasL antibody for 36 h. CM was supplemented with 0.2% FBS to avoid cell death owing to growth factor depletion. Cell survival was further evaluated by MTT assay. Data are mean±S.D., and are representative of three independent experiments performed in triplicates (**P<0.01 when compared with their respective controls). (**c**i) Apoptotic cell death in target HeLa-EGFP cells in heterogeneous system. Histograms for effector and target cell populations alone or in co-culture are shown by annexin V-PE staining using flow cytometry. 1:Untreated THP-1 MΦ 2: Untreated HeLa-EGFP cells; 3: Untreated effector cells (THP-1 MΦ) were co-plated with target EGFP cells; 4: Effector cells (THP-1 MΦ) were treated with MMC for 24 h, washed with medium and target EGFP cells were co-plated; 5: Untreated effector cells (THP-1 MΦ) were co-plated with target EGFP cells and treated with MG132 for 24 h; 6: Effector cells (THP-1 MΦ) were treated with MMC for 24 h, washed with medium, and target EGFP cells were co-plated and then treated with MG132 for 24 h; 7: Effector cells (THP-1 MΦ) were treated with GW9662 for 2 h followed by addition of MMC for 24 h, and then washed, co-plated with target EGFP cells along with treatment of MG132 and GW9662; 8: Effector cells (THP-1 MΦ) were treated with MMC for 24 h, washed and co-plated with target EGFP cells, and further incubated in the presence of MG132 and anti-TRAIL antibodies; are shown with annexin V-PE positive counts and are indicated as percentages of apoptotic cells; are shown with annexin V-PE positive counts indicated as percentages of apoptotic cells. Upper right quadrant represents proportion of apoptotic bystander target EGFP cells. Effector cells were washed with medium three times before co-plating with the target cells. (**c**ii) The bar graph shows annexin V-PE positive cells in the same experiment. Data are mean±S.D., and are representative of three independent experiments (**P<0.01 when compared with their respective controls). (**d**) Western blot analysis for PARP cleavage. Target HeLa cells were pretreated with 5 *μ*M MG132 for 2 h. Thereafter, CM collected from MMC-treated effector cells (HeLa and THP-1 MΦ) was added in the presence or absence of MG132 for an additional 24 h. Whole-cell lysates were prepared to perform western blot analysis. The levels of PARP (p116) and its cleaved product (p85) were detected. *β*-Actin was used as a loading control

**Figure 6:**
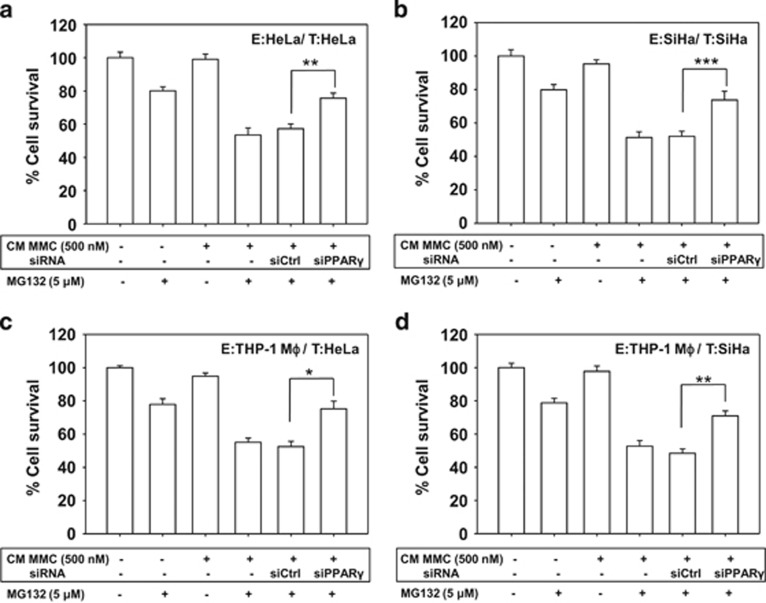
PPAR*γ* knockdown abrogates MMC-induced bystander killing. (**a** and **b**) The effector cells (HeLa, SiHa and THP-1 MΦ) were transfected with control siRNA or PPAR*γ* siRNA for 15 h, fresh medium was added and allowed to grow for the next 15 h. Effector cells (transfected or non-transfected) were treated with 500 nM MMC for 24 h, and then CM medium was collected after 48 h as described in Materials and Methods. Target HeLa (**a**) and SiHa (**b**) cells were incubated with the respective CM in the presence or absence of MG132 for 36 h. CM was supplemented with 0.2% FBS to avoid cell death due to growth factor depletion. (**c** and **d**) Similar experiments were performed in heterogeneous system by incubating target HeLa (**c**) and SiHa (**d**) cells in CM collected from THP-1 MΦ. Cell survival was evaluated by MTT assay. Data are mean±S.D., and representative of experiments performed in triplicates (*P<0.05, **P<0.01, ***P<0.001 when compared with their respective controls)

To mimic the cellular heterogeneity of tumor, the apoptosis-inducing activity of CM collected from MMC-treated THP-1 MΦ was evaluated in target cervical cancer cells. Similar to the observations in the homogeneous system, MG132 treatment enhanced killing of target cells cultured in CM collected from MMC-exposed macrophages compared with control CM (Supplementary Figures 3c and d). Moreover, when CM was supplemented with FasL-neutralizing antibody, bystander cell killing was diminished even in the presence of MG132 (Figures 5a and b). In addition, when HeLa-EGFP cells were co-cultured with MMC-treated THP-1 MΦ and further treated with MG132, a higher percentage of annexin V-PE positive target cells were detected as compared with either condition alone, which is suggestive of MMC-induced contact-dependent bystander killing. These results were consistent with bystander effect observed in homogeneous system (Figures 5ci and cii). Next, to determine whether the bystander cytotoxicity is because of apoptosis, CM transfer experiments were performed. Enhanced PARP cleavage was detected in cells cultured in CM from MMC-treated effector cells in the presence of MG132 in a homogeneous system (HeLa effector cells:HeLa target cells) as well as in the heterogeneous system (THP-1 MΦ effector cells:HeLa target cells; Figure 5d). Finally, to evaluate the role of PPAR*γ* in bystander killing, CM transfer experiments were performed. Effector cells (HeLa, SiHa or THP-1 MΦ) were transfected with control siRNA or PPAR*γ* specific siRNA. Thereafter, the cells were treated with MMC (500 nM) for 24 h and washed twice. CM was collected after an additional 48 h. When the CM collected from PPAR*γ*-knocked down (effector) cells was added to the target cells, the killing of target cells was reduced significantly in both homogeneous (Figures 6a and b) and heterogeneous (Figures 6c and d) systems.

### Co-administration of MG132 and MMC inhibits xenografted tumor progression in mice

**Figure 7:**
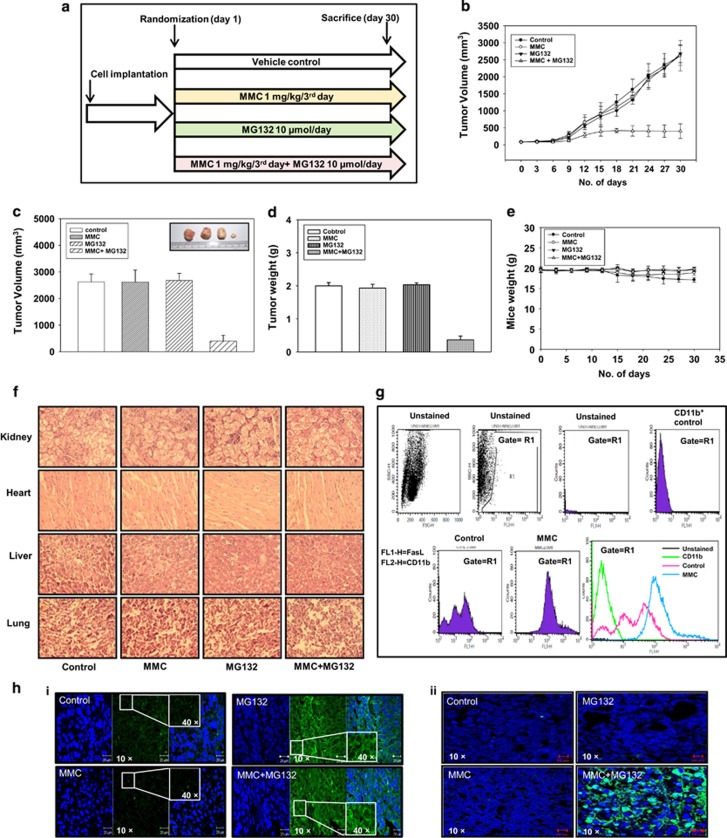
The combination treatment of MMC and proteasomal inhibitor reduces tumor progression in HeLa cells xenografted mouse tumor model. (**a**) Experimental layout of *in vivo* study. HeLa cells (1 × 10^6^ in 100 *μ*l PBS) were injected on the right flank of the mice to form tumors. Tumor-bearing mice were treated with combination of MMC (1 mg/kg/every third day) and MG132 (10 *μ*M/kg/day) as described in Materials and Methods. Control mice were administered with equal volume of vehicle on the same treatment day. (**b**) Tumor progression after drug administration in control and treated mice. (**c** and **d**) Bar graph showing tumor volume and tumor weight in mice at the end of the experiment. (**e**) Changes in body weight in mice during the course of the experiment. (**f**) Histopathological analysis of major vital organs collected from experimental mice. Kidney, heart, liver and lungs were fixed in 4% formaldehyde. The tissues sections were stained with hematoxylin and eosin (H&E; magnification, × 400). (**g**) TAMs were isolated from tumor as described in Materials and Methods. Cells were analyzed for FasL expression by flow cytometry. Cells were dually stained with CD11b (1 : 100) and FasL (1 : 100), and CD11b-positive cells were gated to analyze FasL expression. (**h**) Representative images of immunostained section analysis of Fas (i) TUNEL assay (ii) in tumor tissues of different treatment groups (magnification, × 40 with inset at × 400)

To ascertain our findings *in vivo*, we performed experiments in NOD/SCID mice bearing HeLa xenografted tumor. When tumor volume reached to ~100 mm^3^, mice were divided into four groups (Figure 7a). The mice were treated with MG132 and MMC as described in Materials and Methods. Tumor progression was monitored and followed up during the course of experiment. We observed that combination treatment of MG132 and MMC diminished the tumor progression as compared with either control group or MMC-treated group or MG132-treated group. In mice that received the combination regimen, tumor volume and tumor weight were significantly reduced in comparison to those that received either agent alone (Figures 7b–d). No noticeable change in body weight of mice was detected (Figure 7e), and analysis of hematoxylin and eosin (H&E) stained sections of vital organs (Figure 7f) appeared normal. These observations suggest that MG132 either alone or in combination with MMC does not induce gross toxicity.

Further, to verify the expression levels of FasL in TAMs, macrophages were isolated from xenografted tumors and FasL expression was analyzed by flow cytometry. Interestingly, a significant increase in FasL level was observed in TAMs from MMC-treated groups as compared with those from control group (Figure 7g). To investigate the involvement of bystander elements *in vivo*, tumors were excised and processed for immunohistochemical analysis of Fas expression. The expression of Fas was increased in tumor sections of MG132 alone or combination of MG132 and MMC-treated mice when compared to other groups (Figure 7hi). In addition, tumor sections were subjected to TUNEL assay, which clearly showed more TUNEL-positive cells in tumors from the combination treatment of MMC and MG132 group, indicative of increased apoptotic activity (Figure 7hii).

## Discussion

The strategy of ‘one size fit all' in chemotherapy on the basis of the types and stages of cancer has fallen short in achieving the desired therapeutic index. The role of tumor microenvironment and status of immune system are often not considered toward the outcome of chemotherapy. By engaging immune system of the body, primarily macrophages in chemotherapy, it is possible to enhance the therapeutic outcome. Induction of bystander killing in cancer cells through drug-exposed cancer cells as well as TAMs is likely to be more effective and conceived as an attractive strategy to uproot tumors, which are otherwise less sensitive to chemotherapeutic regimens. Also, it is of translational importance to devise strategies that evoke site-specific BE by using a drug that is known to preferentially accumulate in cervical tumors.^11^ In the present study, we demonstrate that although MMC induces the expression of death ligands in macrophages and cancer cells, bystander killing is not observed. Therefore, restoring Fas level by proteasomal inhibition is crucial to the therapeutic efficacy of MMC.

Effector cells exposed to anti-neoplastic drugs secrete clastogenic factors and death ligands which can act directly or through receptors present on the cell membrane of unexposed cells to trigger a cascade of events culminating in bystander killing. It has been reported that activation of death receptors by specific monoclonal antibodies induces programmed cell death in tumor cells.^32, 33, 34^ We have previously demonstrated that MMC induces FasL-mediated bystander killing in hepatocellular carcinoma cells.^6^ However, the use of MMC in cervical cancer shows inadequate effect owing to its limited efficacy and failing to promote bystander killing. Using various experimental setups, we explored the mechanism of potentiating bystander killing in cervical cancer cells. As bystander killing depends on the drug and cancer cell type, we screened a class of drugs being used clinically in treating cervical cancer. Our data clearly demonstrate that only MMC was capable of inducing expression of death ligands in cervical cancer cells. Proteasomal machinery degrades death receptors, and selective inhibition of proteasomal degradation pathway has been shown to have potent antitumor effect in many cancer types.^35, 36^ With an objective to have sustained expression of Fas on the cell surface of target cells, we pretreated cervical cancer cells with MG132. Although, MG132 treatment alone *per se* caused death in cervical cancer cells, the elevated level of Fas (in target cells) because of blockade in degradation enables the FasL (elevated upon MMC treatment in effector cells) to trigger enhanced killing in target cells. The involvement of FasL was also verified by using FasL-neutralizing antibody in CM transfer experiments. These observations are in agreement with other studies demonstrating that pretreatment of tumors with either bortezomib or depsipeptide enhances sensitivity to TRAIL-mediated killing, primarily by enhancing death receptors DR4 and DR5.^9, 37^

Accessibility of drugs to the irregularly vascularized parts of the tumor is one of the limiting frontiers in cancer chemotherapy. Therefore, bystander killing could overcome these limitations to an ample extent. In this line, we here demonstrate that this sensitization to BE-mediated cell death can be further amplified through the involvement of immune cell-mediated responses. Circulating monocytes in the blood stream are exposed to the chemo-regimen and thus can be exploited to deliver death signals to hitherto inaccessible strata of the tumor. Similar strategies involving virus-laden macrophages have been successfully used to eliminate prostate cancer *in vivo*.^38^ Study by Ho *et al.*^9^ has shown that the PEDF-induced TRAIL expression in macrophages leads to antitumor response *in vivo* and *in vitro*. In yet another study, exposure of TAMs to cyclophosphamide resulted in reduction in tumor progression.^22^ Although, many neoplastic agents are capable of modulating immune responses, the activation of immune responses culminating in promoting tumor cell death, is the yet-to-be-realized target of oncologists.

Our study substantiates that MMC induces expression of FasL in monocytes as well as in differentiated macrophages *in vitro* and *in vivo*. To study macrophage-elicited BE on target cervical cancer cells, heterogeneous system of co-culture and CM transfer experiments were used, but no bystander killing was observed. Previous reports indicate that exposure of cancer cells to proteasomal inhibitors potentiates innate immune response resulting in retarded tumor progression.^36, 37^ We also observed that MMC-induced expression of FasL by macrophages resulted in cytotoxic effect on cervical cancer cells in the presence of proteasomal inhibitor in co-plating and CM transfer experiments. Furthermore, the addition of FasL-neutralizing antibody in CM transfer experiments abolishes the bystander killing facilitated by proteasomal inhibition. These findings clearly suggest the involvement of macrophage-secreted FasL in bystander killing.

As MMC induces expression of FasL in cancer cell as well as in macrophages, the factors involved in transcriptionally regulating FasL in MMC-treated cervical cancer cells and macrophages were sought. DNA damage responses can activate PPAR*γ*, which in turn, regulates the expression of FasL in cancer cells.^27, 39^ We herein demonstrate that knockdown of PPAR*γ* either by siRNA or by an antagonist substantially blocked the ability of MMC to promote FasL expression resulting in reduced bystander killing. These findings confirm that PPAR*γ* has a crucial role in MMC-induced FasL-mediated bystander killing in cervical cancer cells.

The pharmacokinetics of MMC indicates its higher accumulation in cervix supporting the idea of using this drug in cervical cancer therapy.^11^ In addition, MMC in combination with other drugs has been shown to have synergistic effect in cervical cancer cells.^13^ Moreover, our findings are in agreement with other studies demonstrating *in vivo* application of proteasomal inhibitor MG132.^40, 41^ In the present study, we show that MG132 not only potentiates the efficacy of MMC in cervical cancer cells *in vitro*, but also in HeLa xenografted mice with no apparent loss in body weight and minimum overall toxicity to vital organs.

**Figure 8:**
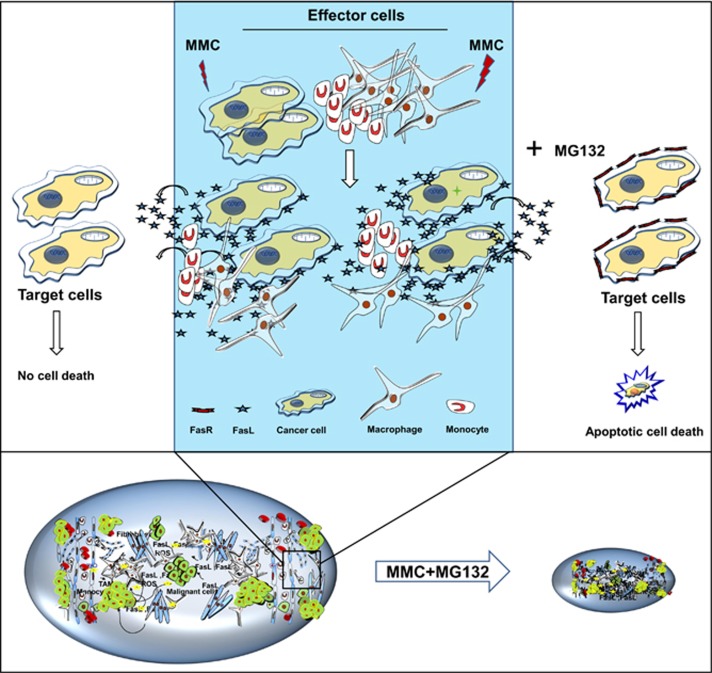
Proposed model for bystander effect. MMC induces expression of membrane bound and secretory forms of death ligands (FasL) in cancer cells as well as macrophages. Restoration of Fas by inhibiting proteasomal degradation facilitates bystander killing of tumor cells and, thus effectively retarding the tumor progression

In conclusion, our study demonstrates that tumor growth retardation is primarily a cumulative effect of MG132-mediated stabilization of Fas levels on tumor cells, and MMC-induced expression of FasL in TAMs and tumor cells (Figure 8). Our findings imply that using proteasomal inhibitors in combination with chemotherapeutic drugs (which can facilitate induction of death ligands in tumor cells and TAMs) could potentially enhance the therapeutic outcome.

## Materials and Methods

### Cell lines and culture conditions

Cervical cancer cell lines (HeLa and SiHa), breast cancer cells (MCF-7 and MDA-MB-231) and monocyte cell line (THP-1) of human origin were obtained from American Type Culture Collection (Manassas, VA, USA), and maintained in our in-house cell repository, National Centre for Cell Science (NCCS), Pune, India. HeLa, SiHa, MCF-7 and MDA-MB-231 cells were routinely cultured in Dulbecco's Modified Eagle Medium (DMEM) and THP-1 cells were maintained in RPMI medium, supplemented with 10% heat inactivated fetal bovine serum (FBS; Sigma-Aldrich, St. Louis, MO, USA), penicillin (100 U/ml) and streptomycin (100 *μ*g/ml; Invitrogen Corporation, Carlsbad, CA, USA) at 37 °C in 5% CO_2_ humidified incubator (Thermo Fisher Scientific, Cleveland, OH, USA).

### Reagents and antibodies

MMC was purchased from Sigma-Aldrich and dissolved in methanol to make a stock solution of 5 mM. MG132 was purchased from Calbiochem (San Diego, CA, USA) and was dissolved in ethanol to make a stock solution of 10 mM. Methylthioazole tetrazolium (MTT; Sigma-Aldrich), was prepared in DMEM without phenol red to make 1 mg/ml working solution. Antibodies against Fas (N-18; sc-714), FasL (c-178; sc-6237), TRAIL (H-257; sc-7877), PPAR*γ* (sc-7196), PARP (sc-7150), *β*-actin (sc-8432), *β*-tubulin (sc-9104), recombinant human FasL (hBA-175; sc-4855), normal rabbit IgG-B (sc-2763), HRP- and FITC-conjugated secondary antibodies were purchased from Santa Cruz Biotechnology (Paso Robles, CA, USA). Annexin V-PE (cat #559763), annexin V-FITC (cat #556570) and antibodies against CD11b (cat #553311), were purchased from BD Biosciences (San Jose, CA, USA). FasL-neutralizing antibody (cat #AB126) and TRAIL-neutralizing antibody (cat #MAB375) were purchased from R&D Systems (Minneapolis, MN, USA) and reconstituted in sterile PBS (phosphate-buffered saline, pH 7.4) to make 1 mg/ml solution.

### MTT assay

HeLa or SiHa cells (7 × 10^3^/well) were plated in 96-well plates and allowed to adhere. After 24 h, cells were treated with or without 5 *μ*M MG132 for 2 h. Thereafter, these cells were treated with two concentrations of MMC (200 or 500 nM) for further 34 h. Viability of cells was measured by MTT assay as described previously.^42^

### Differentiation of THP-1 macrophages (THP-1 MΦ)

THP-1 monocytes were seeded by resuspending them in culture dishes as per experimental requirements in the presence of 20 nM phorbol 12-myristate 13-acetate (PMA) for 18 h. Thereafter, the differentiated THP-1 MΦ were washed with the medium and treated with MMC and/or inhibitors as per the experimental requirements.

### Transfection of small interfering RNA (siRNA)

Subconfluent HeLa, SiHa and THP-1 MΦ were transfected with 100 nM of human PPAR*γ* siRNA (PDsiRNA; Sigma-Aldrich) or control siRNA (Santa Cruz Biotechnology) using Lipofectamine 2000 reagent (Invitrogen Life Technologies, CA, USA) in serum-free medium as per manufacturer's protocol. After 15 h of siRNA transfection, culture medium was replaced with new medium for a 15-h recovery period. Thereafter, cells were treated with MMC (500 nM) for an additional 24 h. Next, cell lysates were subjected to western blotting or the CM was collected for medium transfer experiment.

### Collection of CM and quantification of sFasL

To obtain CM, 7 × 10^5^ cells (HeLa, SiHa and THP-1 MΦ) were plated in 60 mm culture dishes and incubated overnight. Cells were then treated with MMC (500 nM) for 24 h. Subsequently, medium was removed, and cells were washed twice with medium, followed by the addition of phenol-red-free DMEM without FBS. Cells were incubated for an additional 24, 48, 72 or 96 h, and CM was collected. Sandwich ELISA for FasL was performed as reported previously.^6^

### Bystander cell killing in CM

*In vitro* CM transfer experiments were designed to understand whether MMC-treated cells (effector cells) induce bystander killing in unexposed cells (target cells). In all the experiments, MMC-treated HeLa, SiHa or THP-1 MΦ were termed as effector cells, whereas MMC-untreated cells were termed as target cells. The target cells (7 × 10^3^/well) were plated in a 96-well plate and pretreated with MG132 for 2 h before the addition of CM. Thereafter, CM supplemented with or without 5 *μ*M MG132 was added for further 34 h, and MTT assay was performed. MG132 concentration was maintained 5 *μ*M throughout the experiment in medium. To abrogate the activity of FasL in inducing BE, neutralizing FasL antibody (1 *μ*g/ml) was added to the CM along with MG132. In case of CM collected from siRNA-transfected cells, cells were first transfected with control siRNA (siCtrl) or PPAR*γ* siRNA (siPPAR*γ*) as described. Thereafter, cells were treated with MMC (500 nM) for 24 h. Subsequently, medium was removed and the cells were washed twice with medium, followed by the addition of fresh medium. After 48 h, the CM was collected from effector cells, and the target cells were treated with or without MG132 in this CM.

### Immunoblotting and immunofluorescence

Whole-cell lysates were prepared and immunoblotting was performed as described previously.^43^ For immunofluorescence, cells (7 × 10^3^) were plated in multitest slides (MP Biomedicals, Solon, OH, USA) and treated as per the experimental requirements. Next, the cells were fixed, permeabilized and immunofluorescence analysis was performed as described previously.^5^

### Reverse transcriptase (RT) PCR

Cells were treated with MMC as described above, and RNA was extracted from cells as per the manufacturer's instructions (Invitrogen Life Technologies). Synthesis of cDNA and RT-PCR were performed using thermal cycler (Eppendorf, Hamburg, Germany). PCR reactions were performed using the following primers: human Fas sense, 5′-GGGTGAAGAGAAAGGAAGTACAG-3′, human Fas antisense, 5′-CCTTGGAGGCAGAATCATGA-3′ human FasL sense, 5′-CAGGACTGAGAAGAAGTAAAACCG-3′, human FasL antisense, 5′-CTCCAAAGATGATGCTGTG-3′ human *β*-actin sense, 5′-TGACGGGGTCACCCACACTGTGCCCATCTA-3′, and human *β*-actin antisense, 5′-CTAGAAGCATTTGCGGTGGACGATGGAGGG-3′. The annealing temperature used was 58 °C for all sets of primers. PCR products were visualized and photographed using a gel doc (Bio-Rad, Hercules, CA, USA).

### Flow cytometry

The cells (3 × 10^5^) were plated in 35 mm culture dishes, allowed to adhere for 24 h and treated as per the experimental requirements. Thereafter, the cells were collected by trypsinization, pelleted down by centrifugation at 1 500 r.p.m. for 5 min and washed twice with 1 ml PBS. The cells were incubated in PBS containing 10% FBS for 30 min and washed with PBS. Washed cells were suspended in 50 μl of fluorescence-activated cell sorting buffer (2% FBS in PBS, FACS buffer) to which antibody (FasL/IgG control at 1 : 100 dilution; FasL C-178; sc-6237, Santa Cruz Biotechnology) was added. After incubating at 4 °C for 1 h, cells were washed twice with 1 ml of FACS buffer and stained with PE-labeled secondary antibodies (1 : 200 dilution, 30 min). Cells were then washed with 1 ml FACS buffer and resuspended in FACS buffer containing 1% formaldehyde for further analysis. Similar protocol was used to detect the Fas expression on HeLa and SiHa cells. Data were analyzed and mean fluorescence intensity (MFI) was indicated in respective histogram. TAMs were probed with PE-labeled antibody CD11b (at 1 : 100 dilution, 1 h), and then stained for FasL (at 1 : 100 dilution, 1 h; FasL C-178; sc-6237, Santa Cruz Biotechnology), subsequently with FITC-labeled secondary antibodies (at 1 : 200 dilution, 30 min) as described above. Fluorescence intensity was analyzed on FACS Calibur (BD Biosciences), and the data were analyzed using CellQuest Pro software (BD Biosciences).

### Apoptotic death detection by annexin V-PE staining

To evaluate the apoptotic cell death in target cells (HeLa-EGFP and SiHa-EGFP), we performed annexin V-PE staining and the cells were analyzed by flow cytometry. Effector cells (HeLa, SiHa or THP-1 MΦ 3 × 10^5^) were treated with MMC for 24 h. In case of PPAR*γ* inhibition, GW9662 (10 *μ*M) treatment was given 2 h before the addition of MMC to effector cells and incubated for 24 h. Subsequently, the medium was removed and the cells were washed with fresh medium. Target cells (HeLa-EGFP; 3 × 10^5^) were co-cultured overnight with the effector cells (HeLa, or THP-1 MΦ) treated with or without MMC. Co-plated cells were treated with vehicle or 5 *μ*M MG132 for further 24 h. Thereafter, the cells were stained with annexin V-PE according to the manufacturer's protocol (Clontech, Palo Alto, CA, USA). EGFP fluorescence and annexin V-PE positive cells were collected using log amplification and 10 000 events were recorded. Cell population was analyzed using quadrant statistics in an annexin V-PE *versus* EGFP dual parameter histogram, and expressed as percentages. Identical experiments were performed for target SiHa-EGFP cells. Here, we termed the experimental conditions as the following: E/T-EGFP: untreated effector cells were co-plated with target EGFP cells; MMC-E/T-EGFP: effector cells were treated with MMC for 24 h, washed with medium and target EGFP cells were co-plated; E/T-EGFP→MG132: untreated effector cells were co-plated with target EGFP cells and treated with MG132 for 24 h; MMC-E/T-EGFP →MG132: effector cells were treated with MMC for 24 h, washed with medium and target EGFP cells were co-plated and then treated with MG132 for 24 h; GW9662+MMC-E/T-EGFP→GW9662+MG132: effector cells were treated with GW9662 for 2 h followed by the addition of MMC for 24 h, and then washed and co-plated with target EGFP cells along with treatment of MG132 and GW9662; MMC-E/T-EGFP→MG132+anti-TRAIL: effector cells were treated with MMC for 24 h, washed and co-plated with target EGFP cells, and further incubated in the presence of MG132 and anti-TRAIL antibodies.

### Animal studies

Female NOD/SCID mice (aged 5–6 weeks, weight 20±2 g) were procured from Experimental Animal Facility (EAF) at NCCS, Pune, India. All animal experiments were performed as per the requirement and guidelines of the Committee for the Purpose of Control and Supervision of Experiments on Animals (CPCSEA), Government of India, and after obtaining permission from the Institutional Animal Ethics Committee (IAEC). HeLa cells (1 × 10^6^/mouse) in 100 *μ*l PBS were injected subcutaneously on the right flank of mice. When the tumor size reached up to ~100 mm^3^, the animals were randomized into four groups (*n*=5 mice per group). Group one mice were treated with vehicle; group two mice were treated with MMC (1 mg/kg, intraperitoneal on every fourth day); group three mice were treated with MG132 (10 *μ*M/kg/day, intraperitoneal everyday); fourth group mice were co-administered with MG132 (10 *μ*M/kg/day, intraperitoneal everyday) and MMC (1 mg/kg, intraperitoneal on every fourth day) for 30 days. The tumor size was measured every third day using caliper in two dimensions throughout the experiment. Tumor volume (mm^3^) was calculated according to the formula A × B^2^ × 0.52 (A, length; B, width; all parameters in millimeters). At the end of the experiment, the mice were killed by CO_2_ euthanasia. The excised tumors and organs were preserved in 10% paraformaldehyde immediately for immunohistochemical and histological studies.

### Isolation of tumor-associated macrophages (TAMs)

HeLa tumor-bearing mice were killed after 10 days of receiving MMC (third dose). Tumors were harvested, dissected into small pieces and digested with 400 units/ml collagenase type IV, 0.05 mg/ml collagenase type I and 0.01 mg/ml DNase I dissolved in DMEM at 37 °C. Cell suspension pooled from tumors was resuspended in 100 mm culture dishes. After 40 min, adherent cells were characterized through staining with antibodies for CD11b (macrophage marker). FasL expression in these macrophages was analyzed by flow cytometric analysis as described above.

### Immunohistochemistry

Tumors sections (5 *μ*m) were prepared and processed for antigen retrieval as described previously.^42^ Briefly, tissue sections were incubated with 5% FBS for blocking for 1 h. These sections were incubated with primary antibody (at 1 : 100 dilution for overnight at 4 °C) in a humidified chamber. Further, sections were washed and incubated with respective fluorochrome-labeled secondary antibodies (at 1 : 200 dilution for 2 h). Samples were mounted and images were acquired using confocal microscopy (Carl Zeiss, Heidelberg, Germany).

### Detection of apoptosis in tumor sections by TUNEL assay

Analysis of apoptotic cells in tumor tissue was performed by TdT-mediated dUTP Nick End Labeling (TUNEL) assay by using APO-DIRECT (BD Biosciences) following the manufacturer's protocol. Briefly, tumor sections were overlaid with mounting medium containing anti-fade (Santa Cruz Biotechnology). Further, these sections were subjected to confocal microscopy (Carl Zeiss, Heidelberg, Germany).

### Statistical analysis

The data were analyzed using Sigma Plot 12.0 (Systat Software Inc., San Jose, CA, USA), and the values are represented as mean±standard deviation (S.D.). Unpaired student's *t*-test was used to determine the statistical significance of differences between the samples, and *P*-value <0.05 was considered as significant.

## Abbreviations

MMC: mitomycin C
BE: bystander effect
TAMs: tumor associated macrophages
PPARγ: peroxisome proliferator-activated receptor gamma
