# Supplementary material for: Proteasomal inhibition sensitizes cervical cancer cells to mitomycin C-induced bystander effect: the role of tumor microenvironment
Source: Cell Death Dis. 2015 Oct 22;6(10):e1934–. doi: 10.1038/cddis.2015.292 (PMC4632313; doi:10.1038/cddis.2015.292)
Supplement: Supplementary Figures [file cddis2015292x1.pdf]

Title: Proteasomal inhibition sensitizes cervical cancer cells to mitomycin C induced bystander effect: The role of tumor microenvironment

Authors: Shivendra Vikram Singh[1], Amrendra Kumar Ajay[1,2], Naoshad Mohammad[1], Parmanand Malvi[1], Balkrishna Chaube[1], Avtar Singh Meena,[1,3] and Manoj Kumar Bhat[1,*]

Supplementary data

Supplementary figure S1

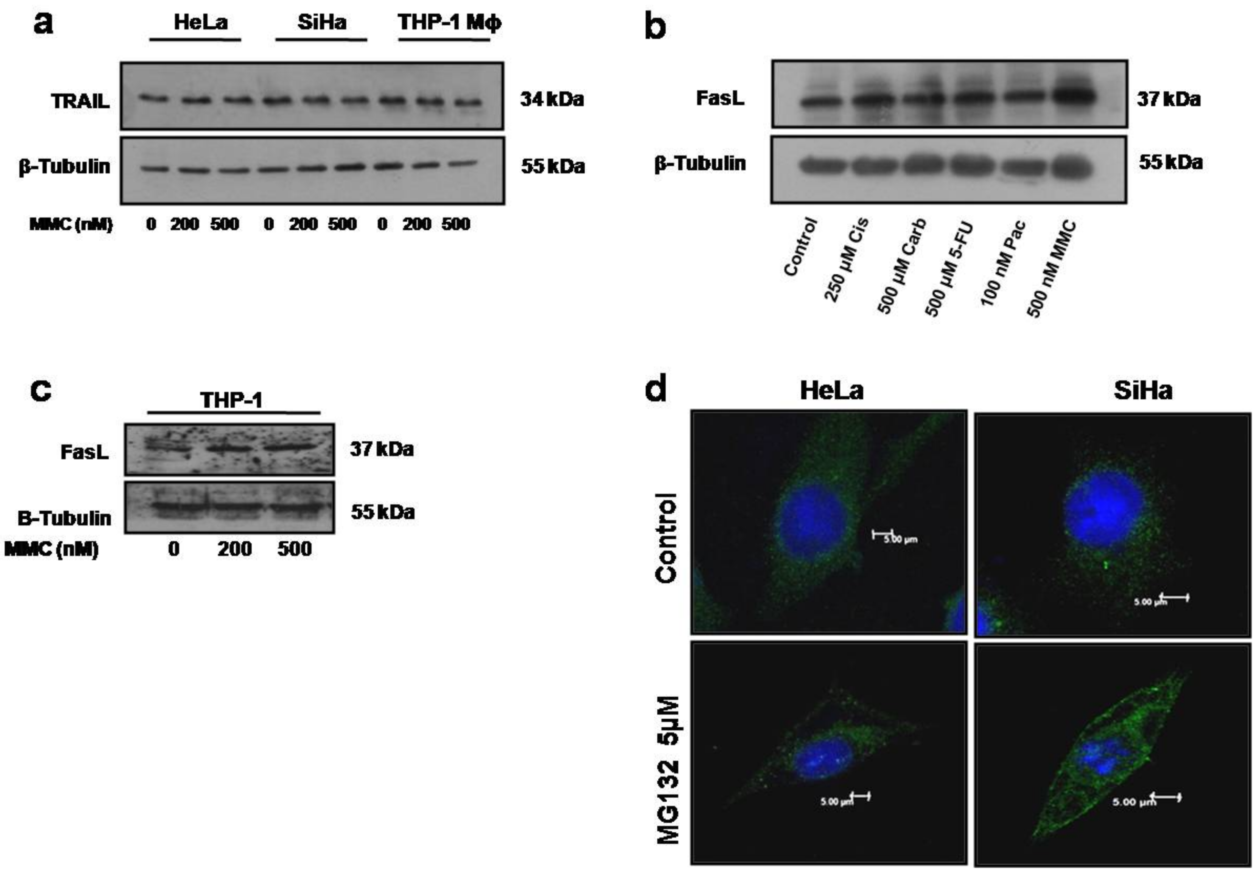

Supplementary figure S1. Expression of factors associated with bystander killing. (a) Western blot analysis of TRAIL. HeLa, SiHa and THP-1 MΦ cells were treated with MMC, and cell lysates were subjected to SDS-PAGE and further probed for TRAIL. (b) Western blot analysis of FasL. HeLa cells were treated with indicated concentrations of cisplatin (Cis), 5-fluorouracil (5-FU), carboplatin (Carb), paclitaxel (Pac) and mitomycin C (MMC). Cell lysates were subjected to SDS-PAGE and further probed for FasL. (c) Immunofluorescence staining of HeLa and SiHa cells. The cells were treated with MG132 for 2 h, and then washed twice with PBS and fresh medium was added for further 24 h. Cells were washed, fixed with 4% paraformaldehyde, permeabilized with 1% triton X-100 and blocked with 5% FBS. Cells were then incubated with anti-Fas primary antibodies (1:100) for 2 h, and subsequently stained with FITC conjugated secondary antibodies (1:200) for 1 h. (d) Western blot analysis of FasL in THP-1 cells. THP-1 cells were treated with MMC, and cell lysates were subjected to SDS-PAGE and further probed for FasL.

Supplementary figure S2

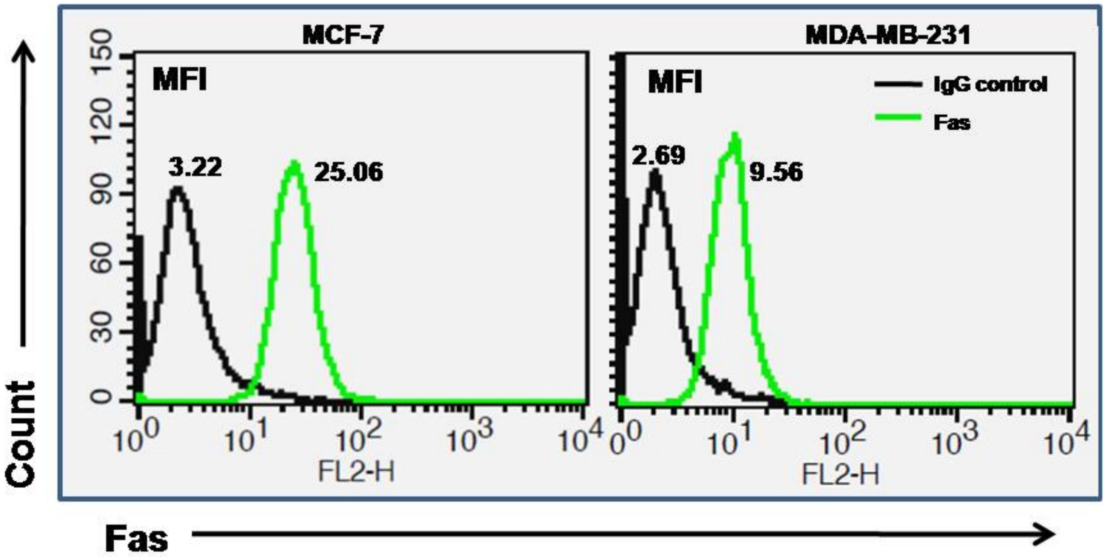

Supplementary figure S2. Fas expression level in breast cancer cells. MDA-MB-231 and MCF-7 cells were trypsinized and washed with PBS. Cells were then probed with Fas antibody or IgG control antibody (1:100) and further with PE-conjugated secondary antibody (1:200). Cells were further washed with PBS, and FasL expression was analyzed by flow cytometry.

Supplementary figure S3

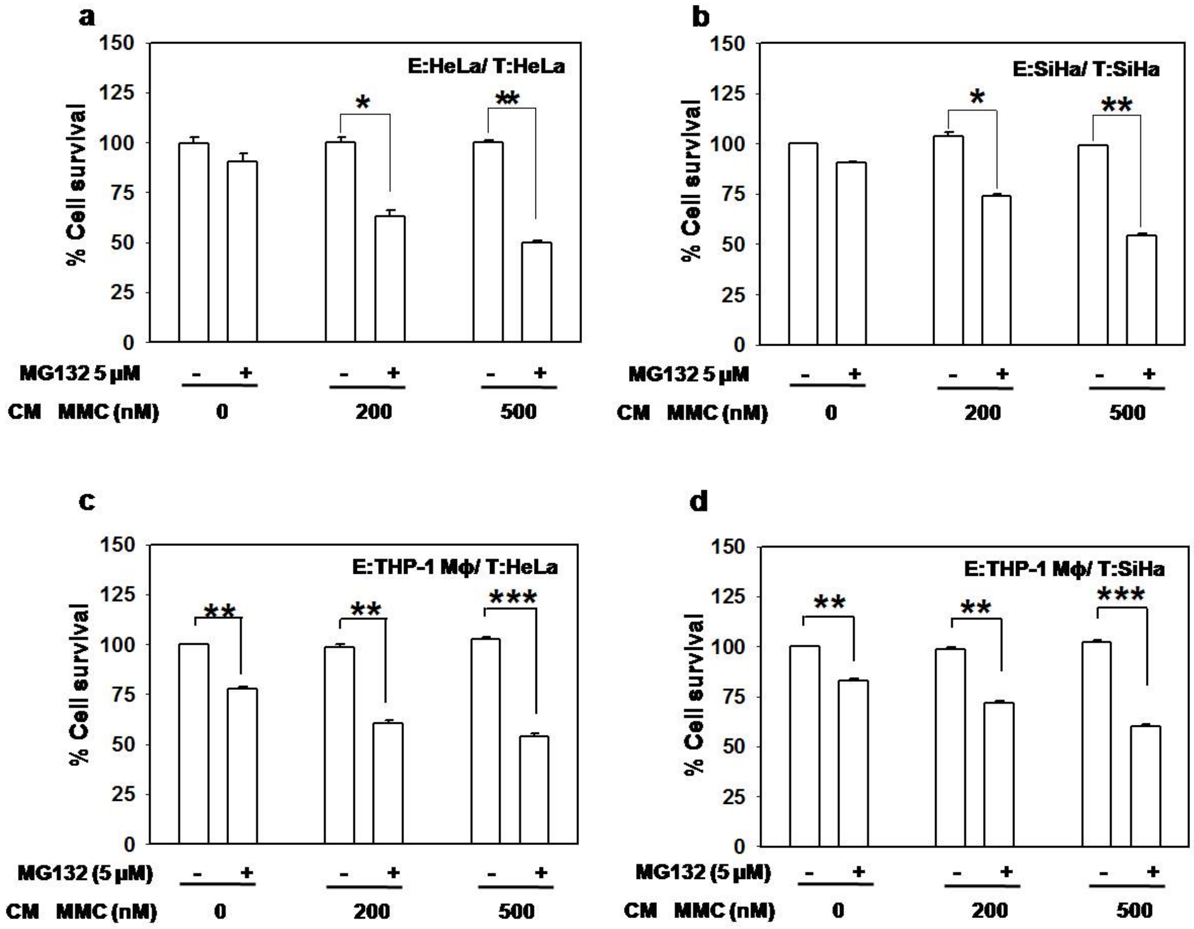

Supplementary figure S3. MMC induced bystander killing of target cells in a dose dependent manner. Secretory form of FasL involved in mediating bystander killing. The effector cells (HeLa and SiHa) were treated with 200 or 500 nM MMC for 24 h and then CM medium was collected after 48 h as described in Materials and Methods. Target HeLa (a) and SiHa (b) cells were incubated with the respective CM in the presence or absence of MG132 for 36 h. CM was supplemented with 0.2% FBS to avoid cell death due to growth factor depletion. Cell survival was further evaluated by

MTT assay. Similar experiments were performed with THP-1 macrophages as effector cells. Target HeLa (c) and SiHa (d) cells were incubated with the respective CM in the presence or absence of MG132 for 36 h. CM was supplemented with 0.2% FBS to avoid cell death due to growth factor depletion. Cell survival was further evaluated by MTT assay. Data are mean ± S.D. and are representative of three independent experiments performed in triplicates (*$p<0.05$, **$p<0.01$, ***$p<0.001$ when compared to their respective controls).
